# Readability Analysis of the Package Leaflets for Biological Medicines Available on the Internet Between 2007 and 2013: An Analytical Longitudinal Study

**DOI:** 10.2196/jmir.5145

**Published:** 2016-05-25

**Authors:** María Ángeles Piñero-López, Pilar Modamio, Cecilia F Lastra, Eduardo L Mariño

**Affiliations:** ^1^ Clinical Pharmacy and Pharmacotherapy Unit Department of Pharmacy and Pharmaceutical Technology University of Barcelona Barcelona Spain

**Keywords:** package leaflet, readability, biological medicines, online health information

## Abstract

**Background:**

The package leaflet included in the packaging of all medicinal products plays an important role in the transmission of medicine-related information to patients. Therefore, in 2009, the European Commission published readability guidelines to try to ensure that the information contained in the package leaflet is understood by patients.

**Objective:**

The main objective of this study was to calculate and compare the readability levels and length (number of words) of the package leaflets for biological medicines in 2007, 2010, and 2013.

**Methods:**

The sample of this study included 36 biological medicine package leaflets that were downloaded from the European Medicines Agency website in three different years: 2007, 2010, and 2013. The readability of the selected package leaflets was obtained using the following readability formulas: SMOG grade, Flesch-Kincaid grade level, and Szigriszt’s perspicuity index. The length (number of words) of the package leaflets was also measured. Afterwards, the relationship between these quantitative variables (three readability indexes and length) and categorical (or qualitative) variables were analyzed. The categorical variables were the year when the package leaflet was downloaded, the package leaflet section, type of medicine, year of authorization of biological medicine, and marketing authorization holder.

**Results:**

The readability values of all the package leaflets exceeded the sixth-grade reading level, which is the recommended value for health-related written materials. No statistically significant differences were found between the three years of study in the readability indexes, although differences were observed in the case of the length (*P*=.002), which increased over the study period. When the relationship between readability indexes and length and the other variables was analyzed, statistically significant differences were found between package leaflet sections (*P*<.001) and between the groups of medicine only with regard to the length over the three studied years (*P*=.002 in 2007, *P*=.007 in 2010, *P*=.009 in 2013). Linear correlation was observed between the readability indexes (SMOG grade and Flesch-Kincaid grade level: r^2^=.92; SMOG grade and Szigriszt’s perspicuity index: r^2^=.81; Flesch-Kincaid grade level and Szigriszt’s perspicuity index: r^2^=.95), but not between the readability indexes and the length (length and SMOG grade: r^2^=.05; length and Flesch-Kincaid grade level: r^2^=.03; length and Szigriszt’s perspicuity index: r^2^=.02).

**Conclusions:**

There was no improvement in the readability of the package leaflets studied between 2007 and 2013 despite the European Commission’s 2009 guideline on the readability of package leaflets. The results obtained from the different readability formulas coincided from a qualitative point of view. Efforts to improve the readability of package leaflets for biological medicines are required to promote the understandability and accessibility of this online health information by patients and thereby contribute to the appropriate use of medicines and medicine safety.

## Introduction

Today, health care professionals are no longer the only source of information for patients on matters related to health because the new information and communication technologies have increased the capacity of patients to seek information independently [1-3]. That is why patient education is growing in importance; in particular, written health-related materials available on the Internet [4], such as package leaflets provided by manufacturers.

One aspect that influences understanding of written information is literacy, which is defined as “using printed and written information to function in society, to achieve one’s goals, and to develop one’s knowledge and potential” [5]. On the basis of this concept, health literacy is defined as “the degree to which individuals have the capacity to obtain, process, and understand basic health information and services needed to make appropriate health decisions” [6]. The capacity of the individual is an important aspect in the definition of health literacy, referring to both innate and acquired skills [7].

Health literacy plays an important role in the evaluation of the online health information [8]. An improvement in health literacy leads to a greater ability of patients to understand how to use medicines appropriately and that is why adequate levels of health literacy can improve medication safety and reduce adverse drug reactions [9,10].

Another aspect that also influences the understanding of written information is its readability, which can be measured using readability formulas. By the 1980s, there were some 200 published readability formulas and a large number of studies that attested to their validity [11]. Nowadays, these formulas are widely applied to measure the readability of health-related written materials [12-15]. It is recommended that the readability level of health-related written materials for end users is not higher than the sixth (age 11-12 years) [7,16] or seventh (age 12-13 years) grade [17].

Among health-related written materials, the package leaflet is “a leaflet containing information for the user which accompanies the medicinal product” [18]. It is very important for the transmission of drug-related information to citizens and it can help to supplement and reinforce the information received from health professionals [19], possibly leading to increased adherence and a consequent decrease in health care costs [4,20].

Several studies have highlighted problems associated with the quality of the information contained in the European model of the package leaflet [21,22]. This is in contrast with current European regulations, according to which the contents of the package leaflet must be clear and understandable, enabling the users to act appropriately, and it must be clearly legible in the official language or languages of the Member State [23]. To achieve this aim, and in accordance with Article 65(c) of Directive 2001/83/EC, in 2009, the European Commission published the Guideline on the Readability of the Labelling and Package Leaflet of Medicinal Products for Human Use, which offers guidance on the writing of the labeling and package leaflet to facilitate understanding and the accessibility of their content [24]. The guidance recommends, among other things, the use of simple words with few syllables and avoidance of long sentences. These explicitly refer to variables included in readability formulas (word length and sentence length).

The European Commission has final authority for authorizing the commercialization of human medicines within the European Union via a centralized authorization procedure. This centralized procedure is compulsory for biological medicines, among others [25]. In 1982, an insulin product became the first recombinant medicine approved for sale [26]. Since then, many biological medicines have been authorized for the treatment of a wide variety of diseases. The biological product market is projected to grow over the coming years and that may affect the introduction both of new products and of existing products for additional indications [27]. In January 2013, there were 171 biological medicines authorized in the European Union [28], 12 of which were biosimilars [29].

In a previous study, the readability levels of package leaflets of some biological medicines (n=33) downloaded from the European Medicines Agency (EMA) website was studied between 2007 and 2010. No variation in readability was found between the two years—the readability of the package leaflets was above the recommended values for health-related materials—and there were differences between the readability levels of the different sections of package leaflets [30]. In view of these findings, it seems important to extend the study to 2013 to see whether the guidance on readability has had an effect in package leaflets until that date.

Thus, the main objective of the current study was to calculate and compare the readability level and length (number of words) of the package leaflets of biological medicines in 2007, 2010, and 2013. We hypothesize that there will be an improvement in the readability of package leaflets, especially in 2013, 4 years after publication of the guidance on readability. We considered 4 years to be sufficient time for manufacturers and marketing authorities to have applied the recommendations in the guidelines. We also wanted to analyze a potential link between readability and length. Moreover, the possible influence of some categorical variables on readability and length was also studied.

## Methods

### Type of Study and Inclusion/Exclusion Criteria

We designed and performed an analytical longitudinal study. The study sample consisted of biological medicine package leaflets that were authorized by the EMA by January 2007 and continued to be authorized in December 2013. Of these, one medicine per active substance was chosen.

The following types of medicines were excluded from the study so as not to introduce bias into the results: vaccines because they are used as a prophylactic measure and, moreover, most are administered by physicians to pediatric patients leading to reduced importance of package leaflets; insulin products because diabetic patients usually know their illness and medicines very well and package leaflet readability is secondary; and botulinum toxin because it was the only biological medicine at the time of the study that contained a toxin as an active substance.

### Data Collection

The package leaflets were downloaded from the EMA website [31] at three different times: January 2007, July 2010, and August 2013. The same pharmaceutical form was chosen in all three years for each medicine.

### Sample Characteristics

As in our previous study [30], the sample of package leaflets (n=36) was divided into five groups depending on the source of the drug [32]: monoclonal antibody (mAb) products, cytokines, therapeutic enzymes, recombinant blood-related products, and recombinant hormones (see Multimedia Appendix 1).

Five of the six sections according to the EMA package leaflet model/template [33] were evaluated: (1) what X is and what it is used for, (2) what you need to know before you take (or use) X, (3) how to take (or use) X, (4) possible side effects, and (5) how to store X. The section (6) “contents of the pack and other information” was excluded because it is considered by patients less important than the other sections [22] and its content was highly similar for all the package leaflets. The “annex” section, which provides information about instructions for use, was also evaluated in the package leaflets where it appeared (10 leaflets in 2007, 11 in 2010, and 12 in 2013).

The evaluated sections of the package leaflets were copied as plain text into individual Microsoft Word 2007 files. Before calculating quantitative variables, the following modifications were made:

1. Titles, subtitles, citations, tables, graphs, images, references, header tables, figure captions, and the brand name of the medicines were deleted.
2. All abbreviations, unit and magnitude symbols, numbers, and acronyms were replaced by their full version because when applying the readability formulas these must be treated as if read aloud [34].
3. Bullets (eg, dashes, numbers, asterisks) were deleted.
4. Compound words and numbers were considered as a single word.

### Quantitative Variables: Readability Indexes and Length

The quantitative variables calculated for the package leaflets were the length (number of words) and three readability indexes: SMOG grade [34], Flesch-Kincaid grade level [35], and Szigriszt’s perspicuity index [36].

The readability indexes were chosen taking into account the following criteria:

1. SMOG grade and Flesch-Kincaid grade level are commonly used in recently published health care literature and they have been validated by different methods [37]. This can make it useful to compare the results obtained with the two formulas.
2. SMOG grade is recommended for use in health-related written materials [1,37,38] because it is the only formula with 100% expected comprehension and is based on more recent criteria for determining reading grade level. For this reason, SMOG grade values are usually higher than Flesch-Kincaid grade level values when both formulas are applied to the same text [37].
3. The qualitative interpretation of Szigriszt’s perspicuity index was designed to assess the readability of written materials in Spanish, which is the language of the package leaflets analyzed in this study.

The SMOG grade formula has as a variable the number of words with three or more syllables, whereas the Flesch-Kincaid grade level and Szigriszt’s perspicuity index use the number of words per sentence and the number of syllables per word. SMOG grade was calculated manually following the author’s instructions, Flesch-Kincaid grade level was calculated using Microsoft Word 2007 software, and Szigriszt’s perspicuity index was calculated following the author’s instructions (before applying this formula, both the number of words and the number of sentences were obtained using Microsoft Word 2007 software and the number of syllables per word was obtained from previously calculated Flesch-Kincaid grade level values).

SMOG grade and Flesch-Kincaid grade level indicate reading grade level (according to the number of years of schooling required after the age of 6 years to understand the text). Thus, sixth-grade level is equivalent to age 11 to 12 years, seventh grade level is equivalent to age 12 to 13, and so on. However, Szigriszt’s perspicuity index has seven qualitative ranges that relate the score obtained and the quality of the text: very easy (85-100), easy (75-85), rather easy (65-75), standard (50-65), rather difficult (35-50), difficult (15-35), and very difficult (0-15) [36]. As the Szigriszt’s perspicuity index score increases, the ease of reading the text also increases. Therefore, the readability is directly proportional to Szigriszt’s perspicuity index and inversely proportional to SMOG grade and Flesch-Kincaid grade level.

The length (numbers of words) of the texts was the fourth variable obtained from the package leaflets because more words on package leaflets can decrease the capacity to find certain information and decrease motivation to read the package leaflet and confidence in using the medicine correctly after reading it [39]. The length was obtained using Microsoft Word 2007 software.

To obtain these variables, the whole text was evaluated in this study to avoid bias that could be introduced in the choice of samples.

### Categorical Variables

The influence of some categorical variables on readability and length was studied. These variables were:

1. Year of downloading the package leaflet (three groups): 2007 (n=36), 2010 (n=36), and 2013 (n=36).
2. Section of package leaflet (six groups): (1) what X is and what it is used for (n=36), (2) what you need to know before you take (or use) X (n=36), (3) how to take (or use) X (n=36), (4) possible side effects (n=36), (5) how to store X (n=36), and annex (n=10 in 2007, n=11 in 2010, n=12 in 2013).
3. Group of medicine according to its source (five groups): mAb products (n=6), cytokines (n=11), therapeutic enzymes (n=4), recombinant blood-related products (n=9), and recombinant hormones (n=6).
4. Date of first authorization of medicine (two groups): 1995-1999 (n=16) and 2000-2002 (n=20).
5. Marketing authorization holder with the real names replaced by letters (five groups in 2007 and four groups in both 2010 and 2013): A (n=3; only in 2007), B (n=3), C (n=5 in 2007, n=4 in both 2010 and 2013), D (n=3), and E (n=3). For this variable, we only considered laboratories with at least three authorized medicines.

For the second variable (section of package leaflet), the values per section were taken into account; for the rest of the variables, the mean of each readability index per package leaflet and the total length of each package leaflet were considered to have a single value per package leaflet.

### Statistical Analysis

Statistical calculations and box plots were performed using Deducer (version R 2.15.0) and R-Commander (version R 3.1.0).

The test of normality used in all the comparative studies was the Shapiro-Wilk test. Taking into account the normality results, the following statistical tests were used to examine the relationship between quantitative and categorical variables: (1) repeated-measures analysis of variance and the Friedman test to compare different groups of each quantitative variable according to package leaflet download date; (2) the Kruskal-Wallis test to compare different groups of quantitative variables according to package leaflet section, source of biological medicine, and marketing authorization holder; and (3) the Mann-Whitney *U* test, to compare different groups of quantitative variables according to date of first authorization of the medicine. Furthermore, the degree of correlation between the readability indexes and length was estimated via the coefficient of determination. A *P* value less than .05 was considered significant in all the hypothesis tests.

Nonstatistical calculations and the other graphs (both line graphs and scatterplots) were generated using Microsoft Excel 2007 software.

## Results

### Effect of Package Leaflet Year on Readability and Length

Table 1 shows readability (mean values for package leaflet) and length results for package leaflet and year studied.

All the SMOG grade and Flesch-Kincaid grade level values exceeded the recommended readability levels for health-related written materials (all SMOG grade and Flesch-Kincaid grade level values were much higher than 6). In addition, all the Szigriszt’s perspicuity index values were less than 75; that is, no package leaflet was easy to understand according to this scale. The respective statistical analysis of the results in Table 1 is presented in Table 2.

**Figure 1:**
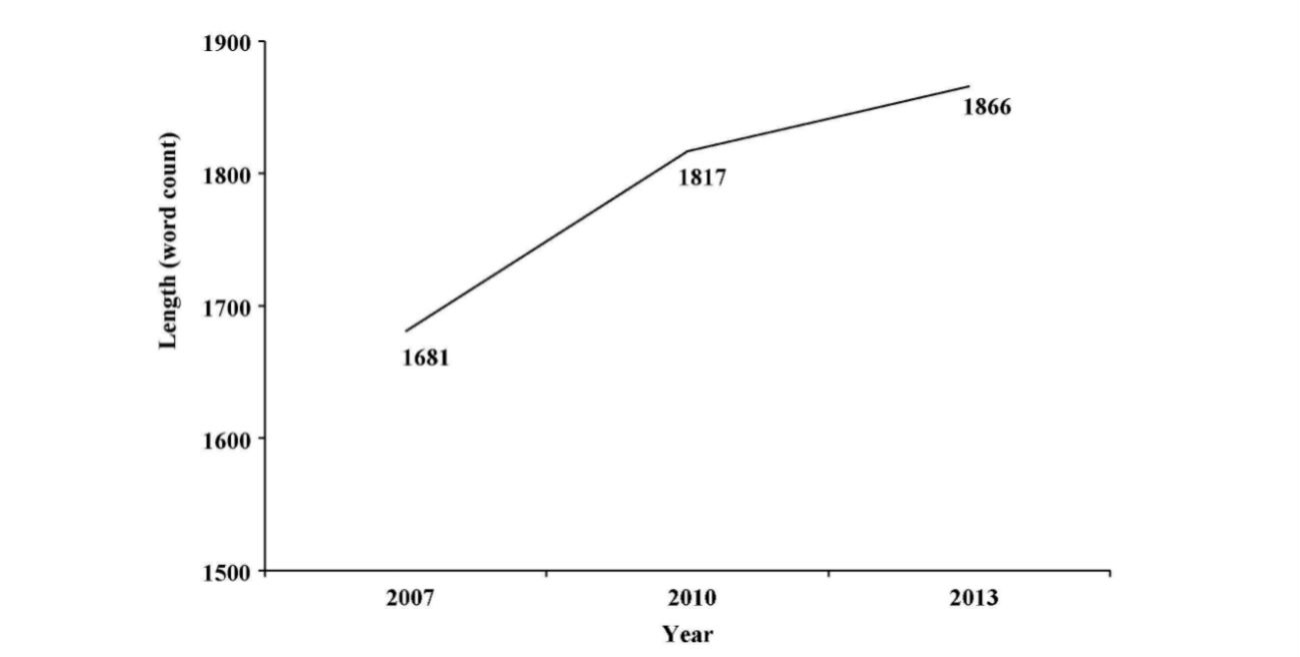
Evolution of the medians of length of the package leaflets studied.

No statistically significant differences were found in the readability values between the three years (*P*=.40 in SMOG grade, *P*=.22 in Flesch-Kincaid grade level, *P*=.20 in Szigriszt perspicuity index), but differences emerged in terms of the length (*P*=.002), with the length of the package leaflets increasing over the 6-year period (Figure 1).

**Table 1:** Mean values of the three readability indexes and total length for package leaflet (n=36) and year studied.

Medicine	SMOG grade, mean (SD)			Flesch-Kincaid grade level, mean (SD)			Szigriszt’s perspicuity index, mean (SD)			Length		
	2007	2010	2013	2007	2010	2013	2007	2010	2013	2007	2010	2013
Agalsidase alfa	16.6 (3.5)	16.6 (3.5)	16.6 (3.5)	16.4 (3.8)	16.3 (4.0)	16.0 (3.6)	58.4 (12.3)	58.9 (13.5)	60.2 (12.6)	905	907	940
Agalsidase beta	18.4 (3.1)	18.0 (5.1)	18.4 (4.8)	19.5 (3.6)	18.5 (6.2)	18.2 (5.6)	45.4 (11.6)	50.5 (21.7)	51.6 (19.3)	986	851	1131
Anakinra	14.8 (1.7)	14.8 (1.7)	15.2 (1.5)	13.3 (1.9)	13.4 (1.8)	13.6 (1.8)	68.4 (8.0)	68.1 (8.1)	67.3 (7.9)	1349	1429	1584
Basiliximab	17.0 (3.6)	17.2 (4.7)	17.2 (4.7)	16.6 (3.8)	16.5 (5.3)	16.7 (5.6)	56.3 (13.5)	58.5 (19.7)	58.0 (20.6)	975	949	970
Choriogonadotropin alfa	16.8 (1.9)	16.8 (1.9)	17.6 (2.6)	16.3 (2.8)	16.3 (2.8)	17.4 (3.6)	56.0 (12.1)	56.0 (12.1)	52.9 (15.6)	1217	1217	1705
Darbepoetin alfa	16.3 (1.8)	16.2 (1.3)	16.5 (1.4)	15.4 (2.3)	15.0 (2.4)	15.5 (2.5)	60.8 (8.9)	62.1 (10.4)	59.9 (10.4)	2326	2125	2324
Desirudin	16.2 (1.3)	17.6 (2.7)	17.6 (2.7)	15.2 (1.6)	16.8 (3.2)	16.8 (3.2)	60.9 (6.7)	55.9 (11.9)	55.9 (11.9)	1253	1167	1167
Epoetin beta	16.6 (2.1)	16.4 (2.7)	16.4 (1.7)	16.2 (2.7)	15.5 (2.6)	15.6 (2.6)	56.6 (10.4)	60.0 (10.5)	59.8 (10.5)	2587	2529	2579
Eptacog alfa (activated)	15.2 (1.8)	16.2 (1.8)	16.2 (1.8)	14.5 (2.1)	15.5 (2.3)	15.4 (2.2)	63.7 (8.2)	62.1 (7.1)	62.7 (6.7)	1158	1426	1431
Eptotermin alfa	18.6 (3.9)	18.6 (3.5)	16.8 (1.6)	18.1 (5.4)	18.1 (5.1)	16.1 (1.8)	52.8 (17.4)	53.7 (15.5)	59.0 (6.0)	1432	1608	1057
Etanercept	17.2 (2.8)	17.5 (2.7)	18.2 (2.9)	16.5 (3.8)	16.5 (3.3)	16.9 (3.4)	58.3 (13.7)	58.6 (12.1)	58.6 (11.4)	3109	3973	4555
Follitropin alfa	17.8 (2.6)	17.8 (2.6)	17.5 (3.1)	17.6 (3.3)	17.6 (3.3)	17.2 (3.8)	51.8 (12.5)	51.8 (12.5)	54.2 (15.4)	2560	2561	3278
Human coagulation factor IX	16.6 (1.9)	16.8 (2.2)	16.8 (2.2)	16.0 (1.9)	16.2 (2.3)	16.2 (2.3)	57.4 (7.3)	57.2 (9.3)	57.1 (9.3)	1737	1874	1874
Human protein C	17.6 (2.9)	17.6 (2.9)	17.8 (3.2)	17.0 (3.4)	17.0 (3.5)	17.0 (3.5)	55.0 (12.0)	55.4 (12.1)	55.5 (12.3)	1761	1760	1768
Imiglucerase	18.2 (2.2)	18.0 (3.5)	18.2 (3.7)	18.0 (2.2)	17.9 (4.1)	17.9 (4.1)	50.4 (8.3)	51.8 (13.7)	51.9 (13.9)	1155	1062	1119
Infliximab	17.8 (2.9)	17.6 (4.8)	17.8 (5.3)	18.3 (3.2)	17.1 (5.6)	17.4 (6.1)	48.4 (12.0)	55.2 (18.7)	54.1 (20.1)	2289	2701	3134
Interferon alfa-2b	17.7 (3.4)	17.8 (3.3)	17.5 (3.1)	16.6 (3.7)	16.6 (3.9)	16.7 (3.8)	57.7 (13.9)	57.5 (14.5)	57.2 (14.4)	3833	4006	4146
Interferon beta-1a	17.6 (2.5)	15.5 (1.8)	15.3 (1.5)	16.8 (2.8)	14.1 (2.2)	14.1 (2.2)	55.4 (10.8)	64.7 (9.3)	64.6 (9.1)	2640	2591	2630
Interferon beta-1b	17.3 (2.7)	17.7 (3.1)	17.7 (3.1)	16.8 (2.7)	17.0 (4.0)	16.9 (4.0)	55.6 (9.8)	55.8 (14.1)	55.8 (14.3)	4868	5463	5450
Lutropin alfa	17.8 (2.9)	17.8 (2.9)	17.8 (3.0)	17.1 (3.4)	17.3 (3.6)	17.1 (3.2)	54.7 (12.4)	53.5 (13.3)	54.0 (11.5)	1794	2110	2346
Moroctocog alfa	18.0 (3.5)	17.6 (1.3)	18.2 (1.9)	17.2 (3.7)	16.5 (2.0)	16.9 (2.2)	54.1 (13.6)	57.0 (8.1)	55.5 (8.7)	1604	2169	2164
Nonacog alfa	17.0 (1.4)	16.8 (1.6)	17.2 (1.9)	16.2 (1.8)	15.7 (2.7)	16.2 (2.7)	58.1 (8.0)	60.5 (10.5)	58.3 (10.4)	2490	2743	2432
Octocog alfa	17.6 (2.9)	17.4 (2.3)	17.4 (2.3)	17.3 (2.6)	17.1 (2.7)	16.8 (2.0)	52.9 (8.6)	53.3 (10.4)	54.8 (7.2)	2029	2403	2249
Palivizumab	15.6 (2.5)	16.6 (3.3)	16.6 (3.3)	15.3 (2.7)	15.9 (4.2)	16.0 (4.3)	61.0 (11.8)	59.3 (18.2)	59.4 (18.2)	548	526	534
Pegfilgrastim	15.5 (1.6)	16.2 (2.6)	15.7 (2.3)	14.3 (2.4)	14.7 (3.0)	14.3 (2.7)	65.4 (9.8)	65.3 (9.7)	66.1 (9.0)	1532	1623	1521
Peginterferon alfa-2a	17.3 (5.4)	15.8 (1.6)	16.2 (2.0)	16.1 (6.5)	14.5 (2.5)	14.8 (2.6)	60.5 (21.2)	64.4 (12.3)	63.8 (11.7)	3005	3545	4151
Peginterferon alfa-2b	17.7 (4.5)	18.5 (5.0)	18.7 (5.8)	17.5 (5.0)	18.1 (6.9)	18.6 (7.3)	53.8 (16.2)	52.6 (18.5)	52.1 (22.2)	3821	4976	5752
Rasburicase	16.2 (1.9)	17.8 (3.4)	18.0 (3.5)	15.7 (3.2)	17.3 (4.1)	17.7 (4.1)	57.4 (14.0)	53.3 (15.8)	52.1 (16.1)	766	714	745
Reteplase	16.8 (2.5)	16.8 (2.5)	16.8 (2.5)	15.9 (2.6)	15.9 (2.6)	15.9 (2.6)	60.4 (9.9)	60.4 (9.9)	60.4 (9.9)	1847	1905	1905
Rituximab	16.2 (2.9)	17.4 (4.5)	18.0 (4.8)	15.0 (3.0)	16.6 (5.3)	17.1 (5.6)	64.0 (10.8)	59.0 (17.3)	57.7 (17.7)	1505	2109	2702
Somatropin	17.0 (2.9)	17.0 (2.9)	17.5 (3.8)	16.7 (3.6)	16.7 (3.6)	17.4 (4.7)	54.9 (15.3)	54.9 (15.1)	53.4 (17.2)	4041	4043	4388
Sulesomab	16.2 (3.3)	16.6 (2.5)	16.6 (3.2)	17.9 (2.2)	17.2 (2.5)	17.1 (3.0)	45.9 (12.2)	51.4 (10.2)	51.6 (11.6)	800	955	962
Tasonermin	19.2 (2.5)	17.8 (3.6)	18.6 (3.8)	19.4 (2.9)	16.9 (4.1)	18.1 (4.3)	47.6 (9.7)	56.9 (18.0)	52.7 (14.5)	2168	1433	1456
Tenecteplase	17.8 (2.8)	18.0 (3.2)	17.4 (1.8)	17.7 (3.0)	17.6 (3.4)	17.0 (2.1)	51.4 (12.4)	51.9 (14.3)	54.6 (10.3)	878	890	1270
Thyrotropin alfa	18.0 (3.6)	17.2 (3.0)	17.4 (3.0)	17.4 (4.4)	16.4 (3.5)	16.7 (3.4)	53.2 (16.8)	56.8 (14.0)	55.7 (13.3)	1219	1240	1286
Trastuzumab	17.0 (3.4)	17.0 (3.7)	17.4 (3.2)	16.5 (3.8)	16.6 (4.0)	17.0 (3.3)	58.7 (13.3)	58.5 (14.3)	56.1 (10.5)	1625	1699	1858

**Table 2:** Descriptive statistics of the three readability indexes and length by studied year and results of the hypothesis tests.

Variable and year		n	Mean (SD)	95% CI	Median	Range	*P*
**SMOG grade**							.40^a^
	2007	36	17.1 (1.0)	16.8-17.4	17.1	14.8-19.2	
	2010	36	17.1 (0.8)	16.9-17.4	17.3	14.8-18.6	
	2013	36	17.2 (0.9)	16.9-17.5	17.4	15.2-18.7	
**Flesch-Kincaid grade level**							.22^b^
	2007	36	16.6 (1.3)	16.2-17.1	16.6	13.3-19.5	
	2010	36	16.5 (1.1)	16.1-16.9	16.6	13.4-18.5	
	2013	36	16.6 (1.1)	16.2-16.9	16.9	13.6-18.6	
**Szigriszt’s perspicuity index**							.20^b^
	2007	36	56.2 (5.2)	54.4-58.0	56.2	45.4-68.4	
	2010	36	57.3 (4.3)	55.8-58.8	56.9	50.5-68.1	
	2013	36	57.1 (4.2)	55.7-58.5	56.0	51.6-67.3	
**Length**							.002^b^
	2007	36	1939 (1031)	1590-2288	1681	548-4868	
	2010	36	2091 (1210)	1682-2501	1817	526-5463	
	2013	36	2238 (1335)	1786-2690	1866	534-5752	

^a^ Repeated-measures ANOVA.

^b^ Friedman test.

### Effect of Package Leaflet Section on Readability and Length

Table 3 and Multimedia Appendix 2 describe the readability indexes and length per package leaflet section in the three years studied. Statistically significant differences can be observed between the four variables and the six sections: (1) what X is and what it is used for, (2) what you need to know before you take (or use) X, (3) how to take (or use) X, (4) possible side effects, (5) how to store X, and (6) annex (*P*<.001).

**Figure 2:**
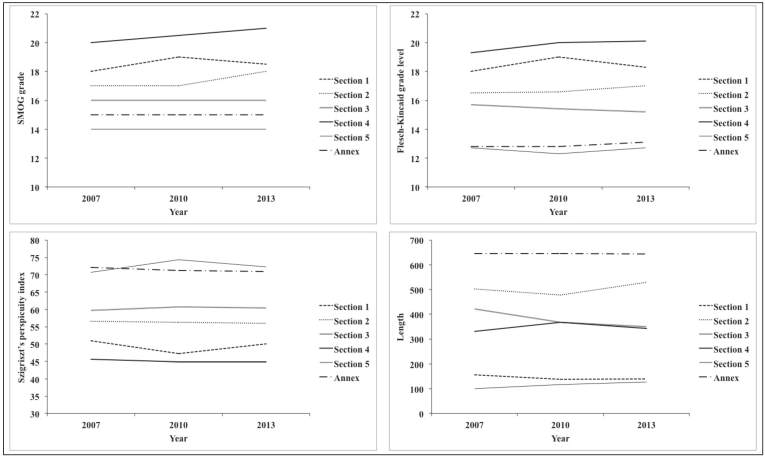
Evolution of the medians by year of SMOG grade, Flesch-Kincaid grade level, Szigriszt's perspicuity index, and length for the package leaflet sections. Section 1: what X is and what it is used for; section 2: what you need to know before you take (or use) X; section 3: how to take (or use) X; section 4: possible side effects; section 5: how to store X; and annex.

These results (Table 3, Multimedia Appendix 2) show the following order of readability of the sections of the package leaflets (from easiest to understand to most difficult): (1) section 5, (2) annex, (3) section 3, (4) section 2, (5) section 1, and (6) section 4. According to the Szigriszt’s perspicuity index medians (Table 3) and the scale of perspicuity level, section 4 was rather difficult, section 1 was standard in 2007 and rather difficult in 2010 and 2013, sections 2 and 3 were standard, and section 5 and the annex were rather easy. Thus, sections 1 and 4 were rather difficult to understand in 2010 and 2013, and the readability of section 4 (the most difficult section) decreased during the period studied (Figure 2).

From the median length (Table 3) of the sections of the package leaflets, the following order was determined from longest to shortest: (1) annex, (2) section 2, (3) section 3, (4) section 4, (5) section 1, and (6) section 5. Section 3 decreased in length during the study years, whereas section 2 increased (Figure 2).

**Table 3:** Descriptive statistics of the three readability indexes and length by package leaflet section and year studied.

Variable, year, and section			n	Mean (SD)	95% CI	Median	Range	*P*^a^
SMOG grade								
	2007							<.001
		1. What X is and what it is used for	36	18.4 (2.1)	17.7-19.1	18.0	15-24	
		2. What you need to know before you take (or use) X	36	17.2 (1.3)	16.8-17.6	17.0	14-19	
		3. How to take (or use) X	36	16.3 (1.4)	15.8-16.7	16.0	14-21	
		4. Possible side effects	36	20.3 (2.6)	19.5-21.2	20.0	15-28	
		5. How to store X	36	13.7 (1.2)	13.3-14.1	14.0	11-16	
		6. Annex	10	15.0 (0.7)	14.5-15.5	15.0	14-16	
	2010							<.001
		1. What X is and what it is used for	36	18.5 (1.8)	17.9-19.1	19.0	15-23	
		2. What you need to know before you take (or use) X	36	17.2 (1.3)	16.8-17.6	17.0	14-20	
		3. How to take (or use) X	36	16.1 (1.2)	15.7-16.5	16.0	14-20	
		4. Possible side effects	36	20.7 (2.8)	19.7-21.7	20.5	15-28	
		5. How to store X	36	13.7 (1.0)	13.4-14.1	14.0	11-16	
		6. Annex	11	14.9 (0.7)	14.4-15.4	15.0	14-16	
	2013							<.001
		1. What X is and what it is used for	36	18.6 (1.9)	17.9-19.2	18.5	15-23	
		2. What you need to know before you take (or use) X	36	17.4 (1.3)	16.9-17.8	18.0	14-20	
		3. How to take (or use) X	36	16.2 (1.2)	15.8-16.6	16.0	14-19	
		4. Possible side effects	36	20.9 (3.3)	19.8-22.0	21.0	16-30	
		5. How to store X	36	13.9 (1.0)	13.5-14.2	14.0	11-16	
		6. Annex	12	14.9 (0.7)	14.5-15.3	15.0	14-16	
Flesch-Kincaid grade level								
	2007							<.001
		1. What X is and what it is used for	36	18.4 (2.7)	17.5-19.3	18.0	14.4-25.6	
		2. What you need to know before you take (or use) X	36	16.4 (1.5)	15.9-16.9	16.5	12.6-18.7	
		3. How to take (or use) X	36	16.0 (1.9)	15.3-16.6	15.7	12.7-20.9	
		4. Possible side effects	36	20.0 (3.0)	19.0-21.0	19.3	13.8-28.9	
		5. How to store X	36	13.0 (1.7)	12.4-13.6	12.7	10.0-16.7	
		6. Annex	10	12.9 (1.0)	12.2-13.6	12.8	11.5-14.9	
	2010							< .001
		1. What X is and what it is used for	36	18.6 (2.3)	17.8-19.4	19.0	14.5-24.8	
		2. What you need to know before you take (or use) X	36	16.6 (1.5)	16.1-17.1	16.6	13.3-19.7	
		3. How to take (or use) X	36	15.5 (1.6)	15.0-16.0	15.4	12.0-19.0	
		4. Possible side effects	36	20.4 (3.4)	19.2-21.5	20.0	13.4-29.6	
		5. How to store X	36	12.2 (1.3)	11.7-12.6	12.3	9.0-16.0	
		6. Annex	11	12.8 (0.9)	12.2-13.4	12.8	11.3-14.1	
	2013							<.001
		1. What X is and what it is used for	36	18.5 (2.2)	17.8-19.3	18.3	14.7-23.2	
		2. What you need to know before you take (or use) X	36	16.7 (1.5)	16.2-17.2	17.0	13.1-20.0	
		3. How to take (or use) X	36	15.6 (1.7)	15.1-16.2	15.2	12.2-19.5	
		4. Possible side effects	36	20.5 (3.9)	19.2-21.8	20.1	14.9-32.6	
		5. How to store X	36	12.5 (1.2)	12.1-12.9	12.7	9.0-15.2	
		6. Annex	12	12.9 (0.9)	12.3-13.4	13.1	11.2-14.1	
Szigriszt’s perspicuity index								
	2007							<.001
		1. What X is and what it is used for	36	49.0 (9.6)	45.8-52.3	51.0	31.9-64.1	
		2. What you need to know before you take (or use) X	36	56.5 (5.8)	54.5-58.4	56.6	44.7-69.5	
		3. How to take (or use) X	36	58.0 (8.7)	55.1-61.0	59.7	29.6-71.3	
		4. Possible side effects	36	44.7 (9.5)	41.5-47.9	45.6	20.0-67.0	
		5. How to store X	36	69.5 (8.5)	66.6-72.4	70.7	43.1-82.0	
		6. Annex	10	71.7 (3.9)	68.9-74.5	72.1	64.1-78.2	
	2010							<.001
		1. What X is and what it is used for	36	48.5 (8.1)	45.8-51.3	47.2	36.4-63.4	
		2. What you need to know before you take (or use) X	36	55.9 (6.0)	53.8-57.9	56.2	43.5-66.1	
		3. How to take (or use) X	36	60.2 (6.9)	57.8-62.5	60.7	41.0-73.4	
		4. Possible side effects	36	44.4 (10.4)	40.9-47.9	44.8	17.8-68.6	
		5. How to store X	36	74.0 (6.5)	71.8-76.2	74.3	54.3-88.8	
		6. Annex	11	72.1 (3.3)	69.9-74.3	71.2	67.4-78.2	
	2013							<.001
		1. What X is and what it is used for	36	48.8 (8.6)	45.9-51.7	50.0	28.3-63.4	
		2. What you need to know before you take (or use) X	36	55.9 (5.9)	53.9-57.9	55.9	42.1-68.3	
		3. How to take (or use) X	36	59.8 (7.5)	57.3-62.3	60.4	39.0-72.6	
		4. Possible side effects	36	44.2 (11.3)	40.4-48.0	44.8	10.7-63.0	
		5. How to store X	36	72.7 (5.6)	70.8-74.6	72.2	58.8-88.8	
		6. Annex	12	71.6 (3.2)	69.6-73.6	70.9	67.4-78.0	
Length								
	2007							<.001
		1. What X is and what it is used for	36	166 (75)	140-191	157	62-433	
		2. What you need to know before you take (or use) X	36	518 (323)	409-628	502	89-1641	
		3. How to take (or use) X	36	498 (351)	379-617	422	89-1331	
		4. Possible side effects	36	399 (236)	320-479	331	113-984	
		5. How to store X	36	102 (39)	88-115	100	32-168	
		6. Annex	10	922 (519)	551-1294	646	481-2114	
	2010							< .001
		1. What X is and what it is used for	36	163 (93)	131-194	138	54-401	
		2. What you need to know before you take (or use) X	36	546 (337)	432-660	478	92-1572	
		3. How to take (or use) X	36	477 (339)	362-592	367	93-1332	
		4. Possible side effects	36	491 (364)	368-614	367	75-1726	
		5. How to store X	36	116 (46)	101-132	116	22-229	
		6. Annex	11	977 (588)	582-1372	645	479-2114	
	2013							<.001
		1. What X is and what it is used for	36	168 (96)	136-201	140	54-399	
		2. What you need to know before you take (or use) X	36	618 (388)	487-750	530	92-1572	
		3. How to take (or use) X	36	471 (327)	361-582	350	88-1244	
		4. Possible side effects	36	532 (426)	388-677	342	75-1976	
		5. How to store X	36	132 (50)	115-149	127	22-268	
		6. Annex	12	947 (572)	584-1310	644	479-2114	

^a^ Kruskal-Wallis test.

### Effect of Source of Biological Medicine on Readability and Length

When comparing readability levels as a function of the source of the medicine, no statistically significant differences were observed between the five groups (mAb products, cytokines, therapeutic enzymes, recombinant blood-related products and recombinant hormones) of package leaflets (SMOG grade: *P*=.44 in 2007, *P*=.31 in 2010, *P*=.61 in 2013; Flesch-Kincaid grade level: *P*=.46 in 2007, *P*=.11 in 2010, *P*=.09 in 2013; Szigriszt’s perspicuity index: *P*=.34 in 2007, *P*=.08 in 2013), except when considering Szigriszt’s perspicuity index in 2010 (*P=*.03). However, differences could be observed between the five groups in the length of the package leaflets in the 3 years studied (*P*=.002 in 2007, *P*=.007 in 2010, *P*=.009 in 2013). These differences in length were mainly due to the difference between therapeutic enzymes and cytokines when applying the Bonferroni posttest for multiple comparisons (Table 4). Cytokines had the longest package leaflets: a median of 2640 words (range 1349-4868) in 2007, 2591 words (range 1429-5463) in 2010, and 2630 (range 1456-5752) in 2013; and therapeutic enzymes had the shortest: a median of 946 words (range 766-1155) in 2007, 879 words (range 714-1062) in 2010, and 1030 (range 745-1131) in 2013.

**Table 4:** *P* values obtained by applying the Bonferroni posttest for multiple comparisons of package leaflet length.

Year and type of medicine		*P* value			
		mAb products	Cytokines	Therapeutic enzymes	Recombinant blood-related products
2007					
	Cytokines	.048	—	—	—
	Therapeutic enzymes	.99	.02	—	—
	Recombinant blood-related products	.99	.06	.20	—
	Recombinant hormones	.99	.99	.10	.99
2010					
	Cytokines	.37	—	—	—
	Therapeutic enzymes	.99	.02	—	—
	Recombinant blood-related products	.99	.47	.11	—
	Recombinant hormones	.99	.99	.10	.99
2013					
	Cytokines	.78	—	—	—
	Therapeutic enzymes	.99	.02	—	—
	Recombinant blood-related products	.99	.20	.03	—
	Recombinant hormones	.99	.99	.38	.99

### Effect of Date of First Authorization on Readability and Length

No statistically significant differences were observed in readability levels and length between the leaflets of medicines authorized in 1995-1999 (n=16) and those authorized in 2000-2002 (n=20) as a function of the year of first authorization of the biological medicine (SMOG grade: *P*=.81 in 2007, *P*=.79 in 2010, *P*=.99 in 2013; Flesch-Kincaid grade level: *P*=.79 in 2007, *P*=.81 in 2010, *P*=.94 in 2013; Szigriszt’s perspicuity index: *P*=.57 in 2007, *P*=.78 in 2010, *P*=.96 in 2013; length: *P*=.90 in 2007, *P*=.95 in 2010, *P*=.90 in 2013). However, differences in the descriptive analysis of the data were observed between the medians of the length of the groups: the medians of length of the medicines authorized in 1995-1999 were higher than those of medicines authorized in 2000-2002 in the three years studied. The medians of length for the medicines authorized in 1995-1999 were 1726 (range 548-4868) in 2007, 2007 (range 526-5463) in 2010, and 2035 (range 534-5450) in 2013, although those of the medicines authorized in 2000-2002 were 1681 (range 766-4041) in 2007, 1730 (range 714-4976) in 2010, and 1813 (range 745-5752) in 2013.

### Effect of Marketing Authorization Holder on Readability and Length

Even though differences were found between groups for the readability indexes in 2007 and for Szigriszt’s perspicuity index in 2010 and 2013 (SMOG grade: *P*=.01 in 2007; Flesch-Kincaid grade level: *P*=.01 in 2007; Szigriszt’s perspicuity index: *P*=.01 in 2007, *P*=.03 in 2010, *P*=.04 in 2013), these differences not were observed in the Bonferroni posttest for multiple comparisons (*P*>.05). This was probably due to a lack of statistical power because the samples were considered too small. For this reason, regarding the comparative study of groups of package leaflets as a function of marketing authorization holder, no statistical evidence was available to show the influence of marketing authorization holder on readability and length.

### Relationship Between Quantitative Variables

The results of studying the relationship between the three readability indexes and length are presented in Table 5.

**Table 5:** Correlations between the three readability indexes and length.

Variable	SMOG grade		Flesch-Kincaid grade level		Szigriszt’s perspicuity index		Length	
	*r*^2 ^	*P*^a^	*r*^2 ^	*P*^a^	*r*^2 ^	*P*^a^	*r*^2 ^	*P*^a^
SMOG grade	—	—	.92	<.001	.81	<.001	.05	<.001
Flesch-Kincaid grade level	—	—	—	—	.95	<.001	.03	<.001
Szigriszt’s perspicuity index	—	—	—	—	—	—	.02	.003

^a^ ANOVA.

**Figure 3:**
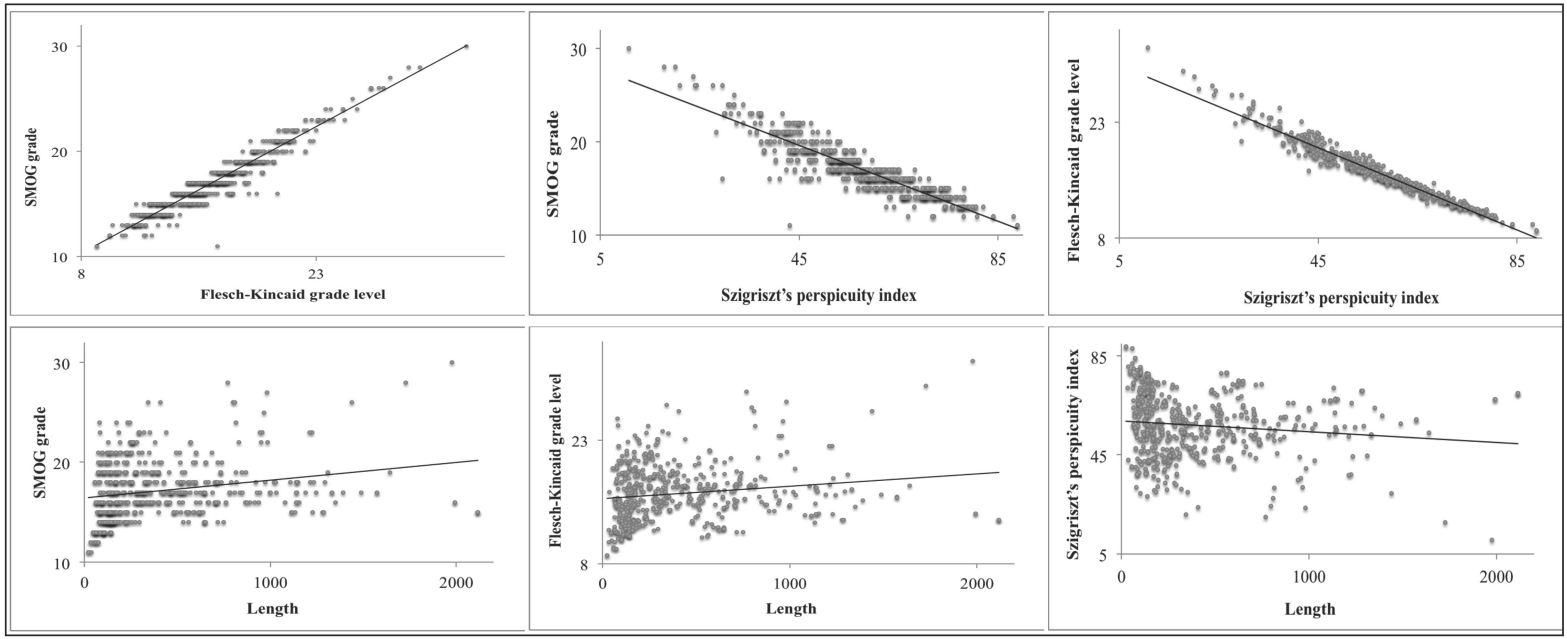
Scatterplots relating SMOG grade, Flesch-Kincaid grade level, Szigriszt's perspicuity index, and length.

Although the *P* value was significant for all pairs of variables, taking into account the coefficient of determination, only a linear correlation between the readability indexes was evident. This correlation can be observed in the respective scatterplots (Figure 3).

## Discussion

### Principal Findings

The objective of this study was to determine the readability and length of package leaflets for biological medicines in three different years. Our results show that none of the package leaflets evaluated met the recommended readability levels for health-related written materials. Therefore, the package leaflets are not in line with European legislation, according to which they must be clearly legible and understandable [23]. This finding could negatively affect patient understanding of the information contained in package leaflets and result in a reduction of adherence intentions [40,41]. A recent study in which patient information materials were evaluated in terms of readability and variety of content found that these materials did not promote health literacy and were only accessible to a proportion of higher skilled patients, which could ultimately increase inequalities in health [42].

Moreover, no improvement in readability was observed over the 6-year period analyzed. This result was not in line with our expectations because the European Commission Guideline [24] recommended the use of words with few syllables and avoidance of long sentences in 2009. In accordance with this guideline, the readability of package leaflets should have increased, but this is not the case.

Furthermore, the number of words in package leaflets increased between 2007 and 2013, which is also a problem for patients. This trend has existed in Europe for a number of years [39]. The observed increase in the length of the package leaflets over the 6-year period studied could be a consequence of prevention policies of the EMA and the time since first authorization that may lead to there being more information related to pharmacovigilance.

When the sections of package leaflets were compared, differences between them were observed in both readability and length in all the years studied. The most difficult sections contain information about therapeutic indications (section 1: what X is and what it is used for) and side effects of the medicines (section 4: possible side effects). This information is considered very important by patients [22] because it highlights the importance of promoting an understanding of the need for medicines and individual risk for side effects when taking medicines [43], and allows them to undertake a rational benefit-risk assessment of their medication [44]. Herber et al [44] stated that *“*package leaflets need to convey potential risk information in a language that is less frightening while retaining the information content required to make informed decisions about the prescribed medication*.”* In contrast, the most understandable section (section 5: how to store X) is considered less important by patients [22].

Regarding the length of sections, the shortest section was section 5 (how to store X) and it was also the most understandable. In contrast, the annex section was the longest section, but it was more understandable than other sections because it has shorter sentences and contains fewer technical terms. Moreover, section 4 (possible side effects) was not the longest section, but it was the least understandable as mentioned previously. This is because in most package leaflets it contains a long list of difficult medical words, which can prevent appropriate interpretation by patients [39].

In relation to the studied correlation between the three readability indexes and total length, only the readability indexes showed an acceptable linear relationship, which was not observed between the readability indexes and length. Thus, we can conclude that the readability of package leaflets is not associated with their length. Fitzsimmons et al [1] assessed the readability of online consumer-oriented Parkinson’s disease information using the Flesch-Kincaid grade level and SMOG grade formulas. They found that webpage length was not associated with readability, suggesting that reading difficulty of websites evaluated was independent of word count. Nevertheless, it is recommendable to reduce the length of package leaflets to make them easier to read and to motivate the patient to access and understand them [1,39]. Indeed, some authors have proposed an alternative template for European package leaflets [45].

### Limitations

Our study has some limitations. First of all, one limitation is the lack of a test of package leaflets according to target patient groups. According to Article 59(3) of Directive 2004/27/EC, package leaflets should reflect the results of user testing to ensure readability. Methods to assess package leaflets using patients have been published [24,46,47], but there are also different studies published in which the results obtained by applying readability formulas and those using user testing have been consistent [48]. Thus, before undertaking a user test or any other form of user consultation [24], the use of formulas can be considered a first step to predict understandability and to initially identify readability problems independently of the type of patient.

A second limitation of this research is the lack of assessment of other characteristics of package leaflets that also improve their readability, such as font, figures, design, and layout [16,24,47,49]. It is important to point out that there are several indirect instruments that assess these characteristics in health-related written materials, such as Suitability Assessment of Materials (SAM) [16] and Patient Education Materials Assessment Tool (PEMAT) [50].

Lastly, not all biological medicines were considered in this study. A sample of 36 biological medicines authorized in both 2007 and 2013 was analyzed taking into account the inclusion and exclusion criteria established. This sample constituted 36 of 61 (approximately 60%) medicines that complied with these criteria in January 2007 and only 36 of 126 (approximately 30%) in January 2013 [28].

Nevertheless, three readability formulas were used (and one of them especially for Spanish) to provide an objective measure of difficulty [11] of reading package leaflets. Moreover, the criteria for obtaining readability indexes have been explained in detail, allowing future comparisons with other studies. Furthermore, to avoid interpretation problems due to a bias in the selection of text samples, all the information contained in the package leaflets was used to calculate the quantitative variables. Finally, this longitudinal study considered adaptation to EU guidelines over a 6-year period of the same health-related written materials.

### Future Implications and Conclusions

Most studies to date have shown that the materials targeted at patients are written with readability levels that make it difficult for the materials to be understood by most people. Package leaflets are this type of written material; therefore, they are no exception to what has been confirmed.

We continue to believe that it would be more than reasonable for applicants and marketing authorization holders, who are responsible for drawing up the package leaflets according to the EMA instructions, to be more conscious of the need to improve the readability of package leaflets together with a decrease of their length. This would increase the usefulness of package leaflets and access to the information they contain by patients.

In the same way, it is advisable for those responsible for drawing up the package leaflets to measure the readability with some of the formulas applied here (which we have shown to be highly correlated), as a method adopted in parallel to direct methods using patients. In this way, taking the measures as soon as possible and, depending on the results, package leaflets could be revised if necessary. Future studies are needed to explain the reasons why the readability of package leaflets has not improved over the years considered in this study, even though the EU legislation and guidelines have changed.

Finally, we suggest an alternative type of written medicine-related information targeted at patients that covers their information needs because the current package leaflet in the EU has proved to be a barrier that has not introduced significant changes in either its readability or length. The alternative written material should be more concise (shorter) than the current package leaflets and it should be written in clear language that is comprehensible for patients.

## Appendix

Multimedia Appendix 1 Biological medicines studied (n=36).

Multimedia Appendix 2 Descriptive statistics of SMOG grade, Flesch-Kincaid grade level, Szigriszt's perspicuity index, and length for each package leaflet section and year studied. Section 1: what X is and what it is used for; section 2: what you need to know before you take (or use) X; section 3: how to take (or use) X; section 4: possible side effects; section 5: how to store X; and annex.

## Abbreviations

EMA: European Medicines Agency
EU: European Union
mAb: monoclonal antibody
