# Supplementary material for: Readability Analysis of the Package Leaflets for Biological Medicines Available on the Internet Between 2007 and 2013: An Analytical Longitudinal Study
Source: J Med Internet Res. 2016 May 25;18(5):e100. doi: 10.2196/jmir.5145 (PMC4899622; doi:10.2196/jmir.5145)
Supplement: Multimedia Appendix 1 [file jmir_v18i5e100_app1.pdf]

Active substance	Date of authorization	ATC[a] code	Therapeutic subgroup
**mAb[b] products** (n=6)			
Sulesomab	14/02/1997	V04D	Diagnostic agents
Rituximab	02/06/1998	L01XC02	Antineoplastic agents
Basiliximab	09/10/1998	L04AC02	Immunosuppressants
Infliximab	13/08/1999	L04AB02	Immunosuppressants
Palivizumab	13/08/1999	J06BB16	Immune sera and immunoglobulins
Trastuzumab	28/08/2000	L01XC03	Antineoplastic agents
**Cytokines** (n=11)			
Interferon beta-1b	30/11/1995	L03AB08	Immunostimulants
Interferon beta-1a	13/03/1997	L03AB07	Immunostimulants
Epoetin beta	16/07/1997	B03XA03	Antianemic preparations
Tasonermin	13/04/1999	L03AX11	Immunostimulants
Etanercept	03/02/2000	L04AB01	Immunosuppressants
Interferon alfa-2b	09/03/2000	L03AB05	Immunostimulants
Peginterferon alfa-2b	29/05/2000	L03AB10	Immunostimulants
Darbepoetin alfa	08/06/2001	B03XA02	Antianemic preparations
Anakinra	08/03/2002	L04AC03	Immunosuppressants
Peginterferon alfa-2a	20/06/2002	L03AB11	Immunostimulants
Pegfilgrastim	22/08/2002	L03AA13	Immunostimulants
**Therapeutic enzymes** (n=4)			
Imiglucerase	17/11/1997	A16AB02	Other alimentary tract and metabolism products
Rasburicase	23/02/2001	V03AF07	All other therapeutic products
Agalsidase alfa	03/08/2001	A16AB03	Other alimentary tract and metabolism products
Agalsidase beta	03/08/2001	A16AB04	Other alimentary tract and metabolism products

Active substance	Date of authorization	ATC[a] code	Therapeutic subgroup
**Recombinant blood-related products** (n=9)			
Eptacog alfa (activated)	23/02/1996	B02BD08	Antihemorrhagics
Reteplase	29/08/1996	B01AD08	Antithrombotic agents
Desirudin	09/07/1997	B01AE01	Antithrombotic agents
Nonacog alfa	27/08/1997	B02BD09	Antihemorrhagics
Moroctocog alfa	13/04/1999	B02BD02	Antihemorrhagics
Octocog alfa	04/08/2000	B02BD02	Antihemorrhagics
Tenecteplase	23/02/2001	B01AD11	Antithrombotic agents
Human coagulation factor IX	03/07/2001	B02BD04	Antihemorrhagics
Human protein C	16/07/2001	B01AD12	Antithrombotic agents
**Recombinant hormones** (n=6)			
Follitropin alfa	20/10/1995	G03GA05	Sex hormones and modulators of the genital system
Thyrotropin alfa	09/03/2000	H01AB01	Pituitary and hypothalamic hormones and analogues
Lutropin alfa	29/11/2000	G03GA07	Sex hormones and modulators of the genital system
Choriogonadotropin alfa	02/02/2001	G03GA08	Sex hormones and modulators of the genital system
Somatropin	16/02/2001	H01AC01	Pituitary and hypothalamic hormones and analogues
Eptotermin alfa	17/05/2001	M05BC02	Drugs for treatment of bone diseases

[a]Anatomical Therapeutic Chemical (ATC).

[b]Monoclonal antibody (mAb).
