# Supplementary figures and images for: Readability Analysis of the Package Leaflets for Biological Medicines Available on the Internet Between 2007 and 2013: An Analytical Longitudinal Study
Source: J Med Internet Res. 2016 May 25;18(5):e100. doi: 10.2196/jmir.5145 (PMC4899622; doi:10.2196/jmir.5145)

2007

2010

2013

SMOG grade

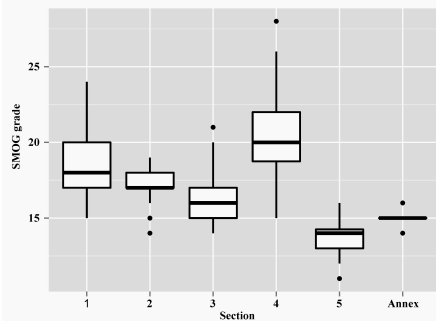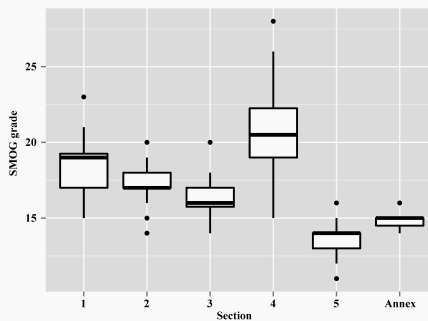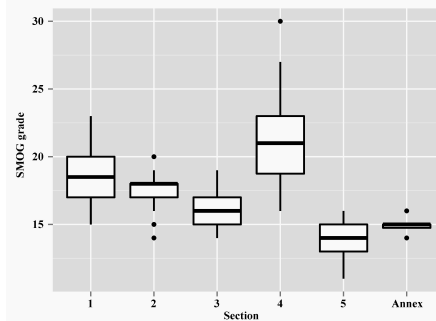

Flesch-Kincaid grade level

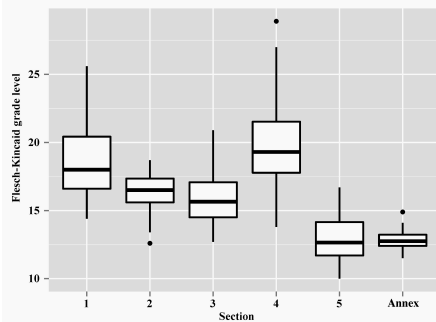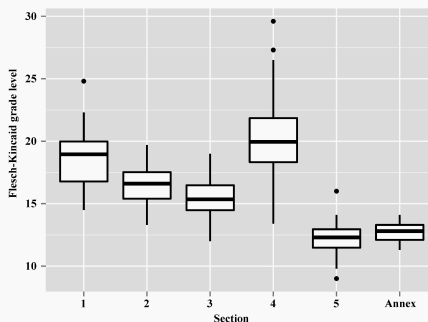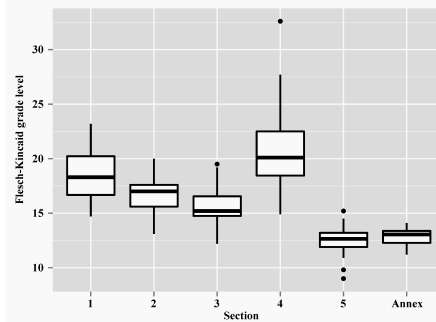

Szigriszt's perspicuity index

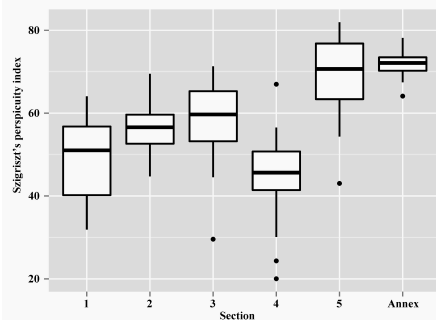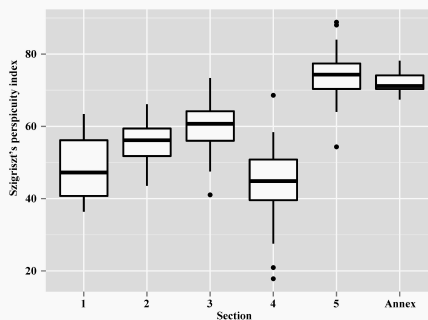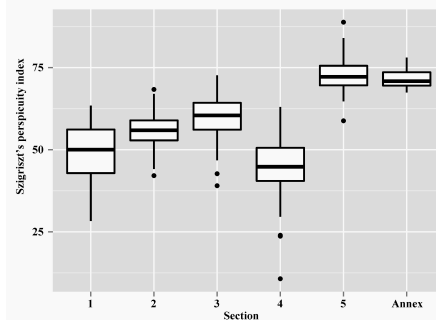

Length

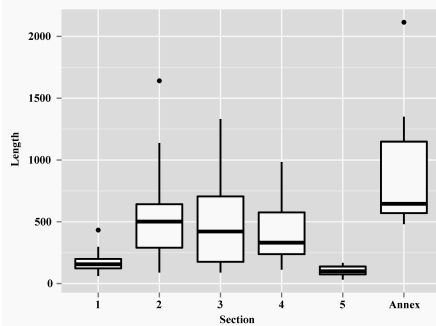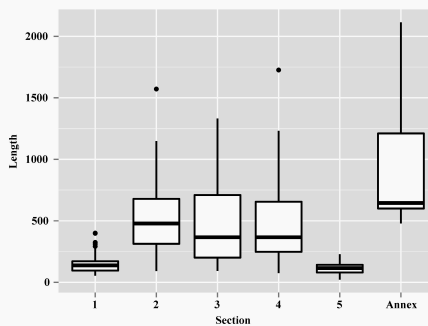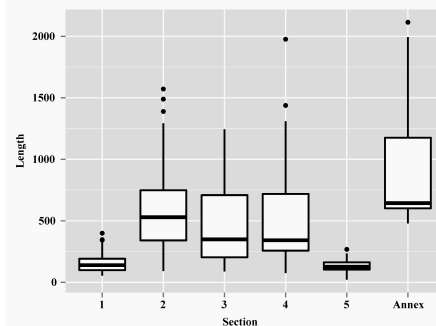

Supplement: Multimedia Appendix 2 [file jmir_v18i5e100_app2.pdf]
